# Glycidyl Methacrylate-Emulsion Styrene Butadiene Rubber (GMA-ESBR)/Silica Wet Masterbatch Compound

**DOI:** 10.3390/polym11061000

**Published:** 2019-06-05

**Authors:** Hyunsung Mun, Kiwon Hwang, Eunho Yu, Woong Kim, Wonho Kim

**Affiliations:** Department of Chemical and Biomolecular Engineering, Pusan National University, Busan 609-735, Korea; ansehdwns10@gmail.com (H.M.); kiwon8348@gmail.com (K.H.); yueh5352@gmail.com (E.Y.); kw65651294@gmail.com (W.K.)

**Keywords:** glycidyl methacrylate (GMA), emulsion styrene butadiene rubber (ESBR), tire tread compound, wet masterbatch

## Abstract

In the tire industry, solution styrene butadiene rubber (SSBR), which can introduce a functional group with good reactivity to silica at chain ends, is used to increase rolling resistance performance by considering fuel economy. However, this is not environmentally friendly because SSBR uses an organic solvent for polymerization, and it is difficult to increase its molecular weight. Functionalized emulsion SBR (ESBR) can solve the problems of SSBR. The molecular weight of ESBR molecules can be easily increased in an eco-friendly solvent, i.e., water. A functionalized ESBR introduces a functional group with good reactivity to silica by introducing a third monomer during polymerization. In this field, glycidyl methacrylate (GMA) has been reported to show the best properties as a third monomer. However, for GMA-ESBR, the viscosity is high and processability is disadvantageous. Therefore, we polymerized GMA-ESBR and manufactured silica compounds to clarify the causes of these problems. In addition, wet masterbatch (WMB) technology, which is a new compound manufacturing method, was applied to manufacture the silica compound, and the physical properties are compared with those of a dry masterbatch. The results clarified the problem of GMA-ESBR, which could be solved by using WMB technology.

## 1. Introduction

In recent years, environmental problems have become a big concern for the tire industry; thus, silica is being used instead of carbon black as a filler to manufacture eco-friendly tires with excellent rolling resistance (RR) performance [1,2]. To improve the RR performance of tires, silica must be well dispersed in a rubber matrix. However, in natural rubber (NR), butadiene rubber (BR), and styrene butadiene rubber (SBR), which are the typically used raw materials for tire treads, there is no polar group and the affinity with silica is poor [3]. There are two main ways to solve this problem: (i) Hydrophobation of the silica surface using a coupling agent and then chemical bonding between the rubber molecules and coupling agents; (ii) facilitating the chemical bonding between rubber and silica by introducing a silica-friendly functional group to the rubber molecules. To improve RR, SSBR is the most widely used rubber in the manufacturing of silica compounds for passenger car tire treads [4]. This is because silica-affinity functional groups can be easily introduced at chain ends due to the nature of anionic polymerization. However, because SSBR is polymerized using an organic solvent, volatile organic compounds (VOCs) are discharged. Furthermore, compared with emulsion SBR (ESBR), which is polymerized through radical mechanism, the molecular weight of SSBR is low and its mechanical properties are poor. Furthermore, ESBR is more eco-friendly than SSBR because it uses water as a solvent. However, chain-end modification in ESBR is difficult because of the characteristics of radical polymerization [5].

To overcome this problem, Thielen [6] introduced a third monomer with a silica-affinity functional group in the polymerization of ESBR. The third monomer used in the polymerization is piperylene, isoprene, vinyl pyridine, hydroxypropyl methacrylate, and acrylonitrile. These third monomers resulted in improved filler-rubber interaction by hydrogen bonding. Therefore, dynamic properties were improved. One of the most effective methods was to polymerize functionalized ESBR by introducing glycidyl methacrylate (GMA). This is because GMA causes improved filler-rubber interaction by chemical reaction with silica. Studies on GMA-ESBR [5,7] suggest that the affinity between rubber and silica was improved by GMA, resulting in improved mechanical properties, dynamic characteristics, and abrasion resistance compared with conventional ESBR. Kim et al. [7] attempted to solve the processability problem of the previous study by controlling the amount of GMA during GMA-ESBR polymerization. Qiao et al. [8] investigated the effect of GMA in GMA-ESBR silica compound without a coupling agent; the GMA-ESBR silica compound showed better physical properties than ESBR silica compound with bis (3-(triethoxysilyl) propyl) tetrasulfide (TESPT), even though TESPT is a silica coupling agent.

GMA-ESBR can be used as a tire tread silica compound for PCR with excellent physical properties, however, it is not used because of its unfavorable processability. Therefore, if the problem of processability can be solved, an excellent tire tread can be manufactured. However, no studies have been tried to solve this problem. In our study, ESBR was also polymerized as a comparative polymer. The silica compounds of GMA-ESBR and ESBR were manufactured through dry masterbatch (DMB) technology, which is a conventional compounding method. To solve the problems, in this study, the silica compound was also manufactured by applying the wet masterbatch (WMB) technique. According to Lightsey et al. [9], WMB is a new method for dispersing silica in emulsion polymers. In addition, modified silica was used for the silica WMB used modified silica, so the silanization reaction is unnecessary in the compounding step; therefore, the temperature can be lowered during compounding [10]. Thus, by using the WMB technology, silica can be dispersed in a rubber matrix without exposure of the functional group of GMA-ESBR to high mixing temperature. This process will prevent the gelation of GMA-ESBR molecules. The physical properties of DMB and WMB compounds were evaluated.

## 2. Experimental

### 2.1. Materials

#### 2.1.1. Materials for Emulsion Polymerization

1,3-Butadiene, surfactant fatty acid, and rosin soap were supplied by Kumho Petrochemical Co. and used without further purification. Styrene and GMA were purchased from Samchun Chemicals CO., Seoul, Korea. A chain transfer agent; tert-dodecyl mercaptan, an initiator; p-menthane hydroperoxide (PMH, 98%), catalysts; sodium formaldehyde sulfoxylate (SFS, 86%), ferrous sulfate (FES, 99%), and ethylenediaminetetracetic acid, and a shortstop agent; diethylhydroxylamine (98%) were purchased from Sigma-Aldrich (St. Louis, MO, USA). Calcium dichloride (CaCl_2_, Daejung Chemicals & Metals Co., Ltd., Siheung, Korea) was used as a coagulant to obtain the rubber.

#### 2.1.2. Materials for the Silica Compounds

In the preparation of the silica compound, used modified silica (7000GR, Evonik, Essen, Germany), which was modified with 10 wt % of bis [3-(triethoxysilyl) propyl] tetrasulfide (TESPT, Evonik, Essen, Germany). As the additives, ZnO, stearic acid (CH_3_ (CH_2_)_16_COOH), N-(1,3-dimethyl-butyl)-N’-phenyl-p-phenylenediamine (6PPD) were used. 1,3-Diphenyl-guanidine (DPG) and N-cychlohexyl-2-benzothiazylsulfenamide (CBS) were used as accelerators in the sulfur vulcanization. Additives, DPG, CBS, and sulfur were purchased from Sigma-Aldrich.

#### 2.1.3. Materials for Analysis of Vulcanizates

Tetrahydrofuran (THF) and n-hexane were used to remove organic substances in the vulcanizates before the swelling experiment, and toluene was used to confirm the crosslink density. All these materials were obtained from Daejung Chemicals & Metals Co., Ltd., Siheung, Korea.

### 2.2. Polymerization of ESBR and GMA-ESBR

Two types of ESBR were synthesized by low-temperature emulsion polymerization by using the formulation shown in Table 1. A high-pressure agitating reactor was made of stainless steel to feed gaseous 1,3-butadiene. The polymerization temperature was controlled by connecting a circulator to the reactor. First, styrene, GMA, water, surfactant, and catalyst were added in the high-pressure reactor; its cap was closed; and air was substituted with nitrogen for 30 min. Then, the initiator was introduced into the reactor by using a syringe, and the gaseous 1,3-butadiene was metered using a chamber and introduced into the reactor by using nitrogen pressure. The temperature of the reactor was maintained at 9 °C, and the total solid contents for the conversion determination were measured using a water drier (MB45, OHAUS, Parsippany, NJ, USA). When the conversion reached 65%, a shortstop agent was added to stop the polymerization.

### 2.3. Conversion Measurement and Latex Coagulation

During the reaction, the polymer was sampled at intervals of 2 h, dried using a water drier, and then the weight ratio was measured and conversion was confirmed by calculating the dry content. A 2 wt % CaCl_2_ aqueous solution was slowly dropped on the prepared latex and coagulated. Then, the coagulated ESBR was dried in a circulating hot air dryer at 50 °C for 24 h.

### 2.4. Characterization of ESBR

The contents of styrene, GMA, butadiene, and vinyl in butadiene were determined by using a nuclear magnetic resonance spectrometer (^1^H-NMR; Varian, Unity Plus 300 spectrometer, Garden State Scientific, Morristown, NJ, USA).

The molecular weight and its distribution were measured using gel permeation chromatography (GPC, Shimadzu, Kyoto, Japan), which consists of a solvent delivery unit, a reflex index detector, and three Stryagel columns (HT 6E, 10 μm, 7.8 mm × 300 mm; HMW 7, 15–20 μm, 7.8 mm × 300 mm; HMW 6E, 15–20 μm, 7.8 mm × 300 mm). Molecular weight was calibrated using polystyrene standard samples. The Mooney viscosity of raw polymers was measured according to ASTM D 1646 using a Mooney viscometer (Vluchem IND Co., Seoul, Korea).

### 2.5. Thermal Stability of ESBR and GMA-ESBR

To determine the thermal stability of GMA-ESBR, ESBR and GMA-ESBR samples were heated for 5 min at 100 °C using a Mooney viscometer (Vluchem IND Co., Seoul, Korea). After that, gel tests were conducted for the virgin samples and heated samples according to ASTM D3616 to determine the increase of gel content caused by the applied heat.

### 2.6. Manufacture of GMA-ESBR/Silica WMB

The surface-modified silica with 10 wt % TESPT was added to distilled water and stirred at 50 °C for 15 min to make silica slurry. Then, the mixture was mixed with GMA-ESBR latex heated at 50 °C and further stirred for 30 min. The WMB was then coagulated with 2 wt % CaCl_2_ aqueous solution, washed once, and dried at 50 °C for 24 h.

### 2.7. Manufacture of Compounds and Vulcanizates

Two-stage kneading was performed by applying the formulation shown in Table 2. The first mixing was performed using a mixer (Kneader, 300cc, Mirae SI Co., Gwangju, Korea) with a fill factor of 0.7. The start temperature was 110 °C, and the end temperature was controlled to 150–155 °C. In the second mixing, sulfur and crosslinking accelerator were added and mixed for 2 min, and sheet-forming was performed by using a two-roll mill (rotor speed ratio of 1:1.1). After the second mixing, the specimens were crosslinked during the optimum vulcanization time (*t*_90_) in a 160 °C with 3.5 bar pressure by using a hydraulic high-temperature press. The detailed mixing procedure is described in Table 3.

### 2.8. Experimental Methods

#### 2.8.1. Payne effect

The Payne effect was measured to confirm the silica dispersion. Rubber process analyzer (RPA 2000, Alpha Technologies, Hudson, OH, USA) was used according to ASTM D 8059.

#### 2.8.2. Cure Characteristics

Cure characteristics, such as the minimum and maximum torque values and the optimum vulcanization time (*t*_90_), were determined by using a moving die rheometer (MDR, Myung Ji Co., Seoul, Korea). The measurement was performed using a silica compound with a vibration angle of ± 1° and a temperature of 160 °C for 30 min. By using the optimum vulcanization time, vulcanization was performed in a 160 °C press.

#### 2.8.3. Analysis of the Vulcanizate Structure

The crosslinked specimen was cut into a size of 10 mm × 10 mm, and the organic additives in the specimen were removed by immersing the specimen in THF and n-hexane as a solvent for 2 days and 1 day, respectively, followed by drying for 1 day at room temperature. The specimen from which the additives were removed was swollen with toluene for 1 day and then weighed; the following equation was used to calculate crosslink density *v* (mol/g):

(1) $$v=\frac{1}{2M_{c}}=-\frac{\ln\left(1-V_{1}\right)+V_{1}+xV_{1}^{2}}{2\rho_{r}V_{0}\left(V_{1}^{\frac{1}{3}}-\frac{V_{1}}{2}\right)}$$

where *M_C_* is the average molecular weight between the crosslink points (g/mol), *V*_1_ is the volume fraction of rubber in the swollen gel at equilibrium, *V*_0_ is the molar volume of solvent (cm^3^/mol), *ρ_r_* is the density of the rubber sample (g/cm^3^), and *χ* is the polymer–solvent interaction parameter.

#### 2.8.4. Mechanical Properties

A universal testing machine (UTM, Model: KSU-05M-C, KSU Co., Ansan, Korea) was used to measure the modulus, tensile strength, and elongation at break. The dumbbell specimens were prepared according to ASTM D412. The measurement speed was 500 mm/min.

#### 2.8.5. Abrasion Loss

The specimens were prepared according to DIN 53516 for wear testing. After the initial weight of the specimen was measured, the specimen was abraded for 40 m at a speed of 40 rpm with 5N of load using a Deutsche Industrie Normen (DIN) abrasion tester, and the specimen weight was measured again to measure weight loss.

#### 2.8.6. Dynamic Viscoelastic Properties

By using a dynamic mechanical thermal analyzer (DMTA, EPLEXOR 500N, GABO, Ahlden, Germany), the three vulcanizates were measured at a strain of 30 µm and a frequency of 10 Hz. The measurement was conducted in the tension mode, at a temperature increase of 3 °C/min, and a temperature range of –80 to 70 °C.

## 3. Results and Discussion

### 3.1. Characterization of ESBR and GMA-ESBR

**Figure 1:**
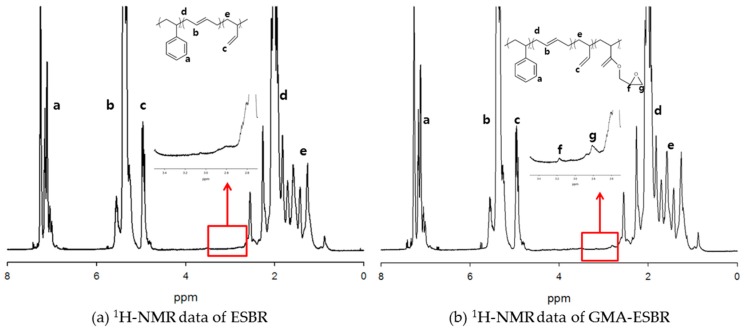
^1^H-NMR data of ESBR and GMA ESBR.

The molecular weights of the polymerized ESBR and GMA-ESBR were determined by using the GPC, and the micro structure was confirmed through ^1^H-NMR, shown in Figure 1. The contents of styrene, vinyl in butadiene, and GMA of GMA-ESBR were calculated by considering the molecular weight of the monomer. The results of Mooney viscosity of each polymer were as follows: ESBR and GMA-ESBR were 93 and 124, respectively, so the viscosity of GMA-ESBR was higher. In general, the Mooney viscosity is greatly influenced by the weight average molecular weight [11]. However, because the difference in the Mooney viscosity between the GMA-ESBR and ESBR was considerable compared to the difference of weight average molecular weight, additional experiment for thermal stability of GMA-ESBR was conducted to determine the cause of the large difference in Mooney viscosities. The detailed results of the GPC and NMR measurements are shown in Table 4.

### 3.2. Thermal Stability of ESBR and GMA-ESBR

**Figure 2:**
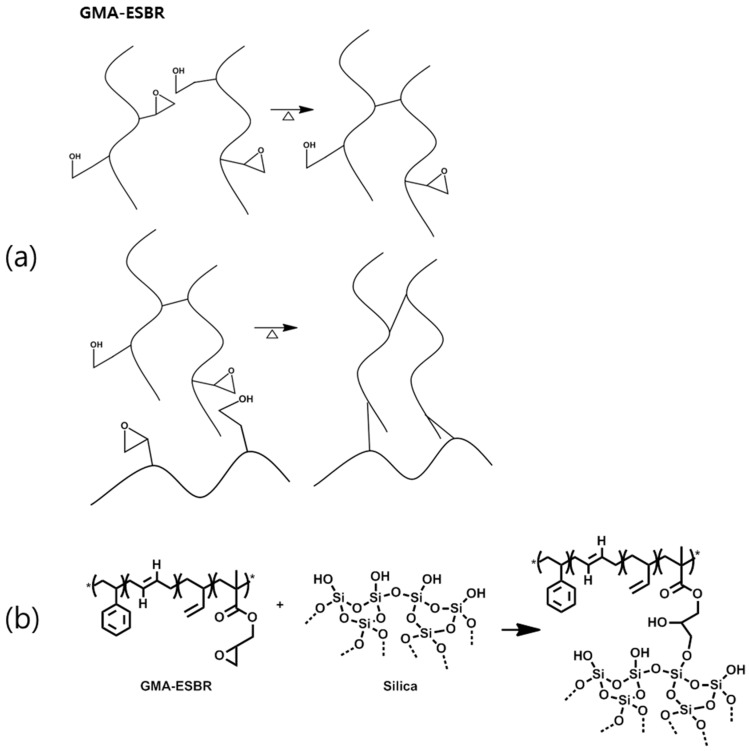
Suggested mechanism of (**a**) gelation based on epoxy ring opening and (**b**) silica-GMA coupling reaction.

Another experiment was conducted to determine why GMA-ESBR has a much higher Mooney viscosity than the ESBR. As shown in Table 5, when GMA-ESBR was heated for 5 min at 100 °C, which is the measurement condition for the Mooney viscosity, the gel contents were confirmed to greatly increase. Thus, a small amount of hydroxyl groups was formed by the opening of the epoxy rings; after that, some of the epoxy rings of the GMA are served to form gels through the chemical reaction with hydroxy group under conditions of Mooney-viscosity measurement [12]. The detailed results are shown in Table 5. These results indicate that GMA can form a gel without reacting with silica at the beginning of the DMB manufacturing process. It is expected that this problem can be solved by manufacturing a silica compound using a WMB technology, which coagulates rubber latex and silica in a 50 °C water phase. In this case, even if the epoxy ring of GMA is opened, the filler–rubber interaction is expected to be great, and less gel formation is expected because the number of silanol groups on the silica surface is larger than the number of epoxy groups of GMA. The expected mechanisms of the gelation caused by the epoxy-ring opening of GMA and the reaction mechanism of the GMA epoxy ring and silanol group on the silica surface are shown in Figure 2.

### 3.3. Silica Dispersion

**Figure 3:**
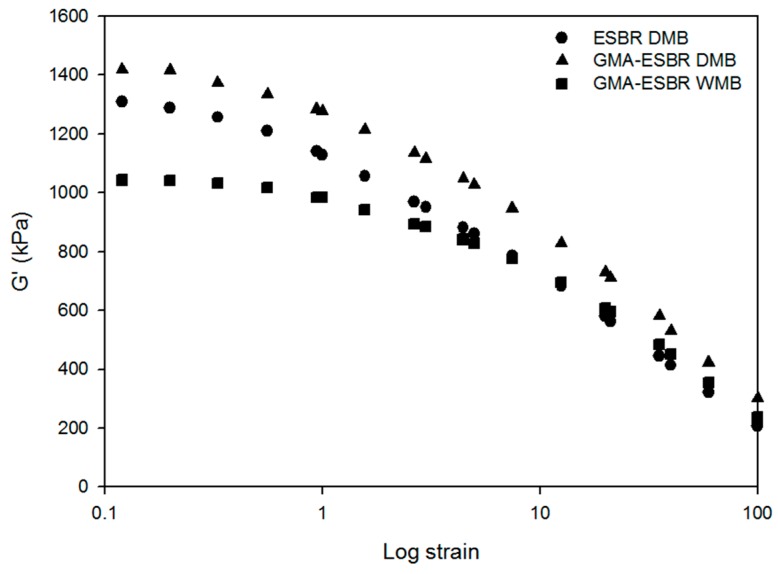
Storage modulus as a function of strain for the ESBR DMB, GMA-ESBR DMB, and GMA-ESBR WMB compounds.

In the Payne effect (ΔG′), storage modulus is decreased by increasing strain, due to the decreased filler-filler interaction. Low values of Payne effect can be obtained, when the silica dispersion is good. The Payne effect of the ESBR DMB, GMA-ESBR DMB, and GMA-ESBR WMB compounds are shown in Figure 3. The results show that the silica dispersion of GMA-ESBR WMB compound is the best. As shown in Table 6, the ΔG′ value of the GMA-ESBR DMB compound is lower than that of the ESBR DMB compound though the value of G′ at low strain is high. This is because the GMA-ESBR DMB compound has high value of G′ at high strain due to gelation. Therefore, it is difficult to clearly compare the silica dispersion of GMA-ESBR DMB compound by Payne effect. However, GMA-ESBR WMB compound has low G′ values in low and high strains, respectively, than GMA-ESBR DMB. Therefore, the gelation problem is resolved through WMB technology.

### 3.4. Analysis of Curing Characteristics and Crosslink Structure

**Figure 4:**
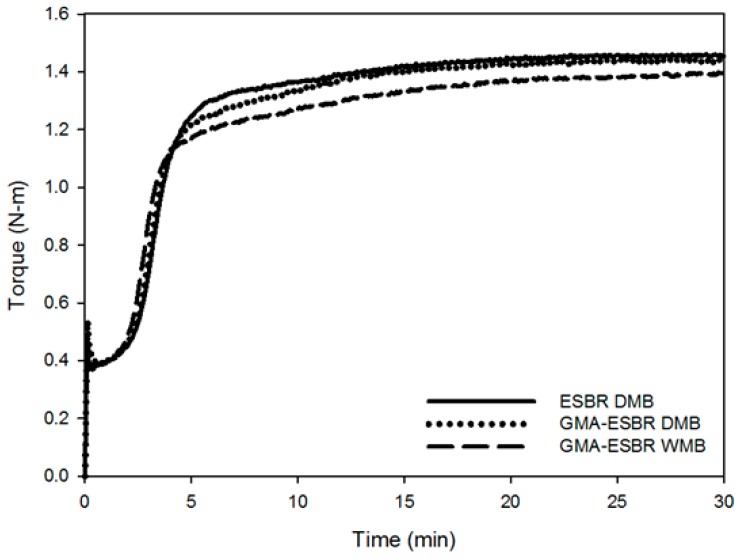
Cure characteristics of ESBR and GMA-ESBR compounds.

The cure characteristics of the ESBR DMB, GMA-ESBR DMB, and GMA-ESBR WMB compounds are shown in Figure 4. The *T*_max_ value of GMA-ESBR compounds were confirmed to be low. In general, the *T*_max_ value is strongly influenced by the crosslink density [13] and filler dispersion in the compound [14]. For the GMA-ESBR compound, the bond strength between the filler and rubber is stronger than that of the ESBR compound. Thus, the agglomerate size should be decreased, i.e., dispersion should be improved. Also, the torque value of GMA-ESBR WMB is lower than that of GMA-ESBR DMB. This is because the silica dispersion of GMA-ESBR WMB is better and processability is improved. Therefore, the WMB technology improves the processability problem of GMA-ESBR. The results of crosslink density measurement are shown in Table 7 together with the evaluation results of the cure characteristics. The results confirmed that the crosslink densities of the GMA-ESBR compounds were higher than that of the ESBR compound. Although the crosslinking densities of the GMA-ESBR compounds are higher than that of the ESBR DMB compound, it can be confirmed that the GMA-ESBR compounds were more dispersed than the ESBR compound because their *T*_max_ values are smaller. In addition, the crosslink densities of the GMA-ESBR DMB and WMB compounds are significantly different. In the early stage of the GMA-ESBR DMB manufacturing process, only the GMA-ESBR was put into the mixer and a gel was formed by the temperature during the initial mixing stage; it is expected that the *V*_1_ value was increased in the analysis of the crosslinked structure by the gel formation. This can be confirmed in the subsequent evaluation of mechanical properties.

### 3.5. Mechanical and Abrasion Properties

**Figure 5:**
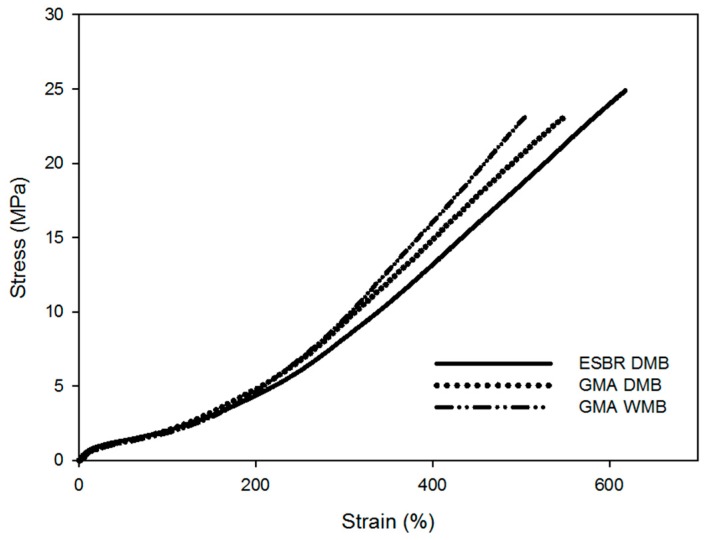
Mechanical properties of ESBR and GMA-ESBR vulcanizates.

Figure 5 shows the results of the mechanical properties of the ESBR DMB, GMA-ESBR DMB, and GMA-ESBR WMB compounds. Although the crosslink density of the GMA-ESBR DMB compound was the highest, the modulus at 300% elongation in the stress–strain curve was the highest in the GMA-ESBR WMB compound. As the modulus is most influenced by the crosslink density [15], the actual crosslink density is considered to be higher for the GMA-ESBR WMB compound. Therefore, the crosslink density of the GMA-ESBR DMB compound is considered to be the result of gel formation during the process of mixing rubber, as previously mentioned. Further, the M_300%_ value of the GMA-ESBR WMB vulcanizate is larger than that of the ESBR DMB vulcanizate. It is considered that the interaction between silica and rubber is strengthened by GMA. Further, the elongation at break is shortened as the crosslink density increases. The GMA-ESBR WMB vulcanizate has the lowest elongation at break, which is the opposite trend as the modulus. Finally, the value of M_300%_/M_100%_ indicates the reinforcement of the filler [16], which has the highest value in the GMA-ESBR WMB vulcanizate, this is due to the epoxy ring reacted with silica without forming a gel. The wear resistance characteristics agreed with the tendency of the M_300%_/M_100%_ value. The wear resistance of the GMA-ESBR WMB vulcanizate, which has the most reinforcing effect of the filler, showed the best performance. The detailed results are shown in Table 8 together with the measurement results of the mechanical properties.

### 3.6. Dynamic Viscoelastic Properties

**Figure 6:**
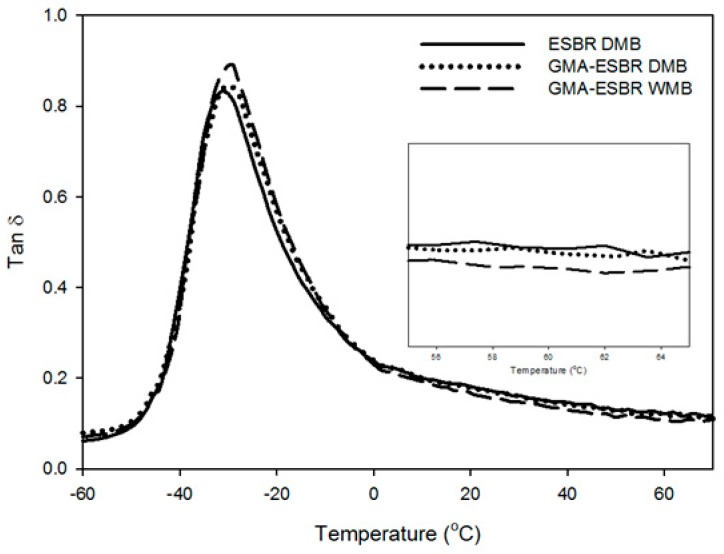
Temperature-dependent tan δ of ESBR DMB, GMA-ESBR DMB, and GMA-ESBR WMB compounds.

The results of dynamic viscoelastic properties are shown in Figure 6 and Table 9. The difference in the T_g_ of the compound using ESBR and GMA-ESBR is due to the difference crosslink density. The tan δ at T_g_ value is a measure of the degree of dispersion of the filler. The larger the value, the better the silica dispersion. As with the previous Payne effect results, the silica dispersion of GMA-ESBR WMB compound was the best. In addition, the degree of silica dispersion of GMA-ESBR DMB, which was difficult to analyze clearly by Payne effect, is superior to that of ESBR DMB. Compared with the ESBR DMB vulcanizates, the tan δ value at 60 °C of the GMA-ESBR WMB vulcanizate is superior, i.e., 0.123 vs. 0.108. This is due to the chemical bonding between GMA in the rubber molecules and silica, resulting in improved filler–rubber interaction [5]. The comparison of the GMA-ESBR DMB and WMB vulcanizates showed that the GMA-ESBR WMB vulcanizate showed better RR performance. This is because, during the early stage of mixing, the epoxy ring of GMA in the GMA-ESBR DMB compound reacts with the opened epoxy ring, thereby reducing the reactive site with silica. Therefore, the GMA-ESBR WMB compound showed the best RR performance, indicating that the problem of gel formation by GMA-ESBR can be solved by using the WMB technology.

## 4. Conclusions

This study confirmed that GMA-ESBR can form a gel when heated, resulting in higher compound viscosity and lower processability. GMA-ESBR was predicted to have a better filler–rubber interaction if reacted with silica rather than GMA when the epoxy ring is opened. Therefore, a silica compound was prepared by applying WMB technology, which can coagulate the rubber latex and silica through a relatively uniform dispersion in the rubber latex at low temperatures. Furthermore, the silica compound was manufactured by existing DMB technology, and the properties were compared and evaluated. The results confirmed that the GMA-ESBR silica compound exhibits superior physical properties to the ESBR silica compound, and the GMA-ESBR WMB compound shows better physical properties than the GMA-ESBR DMB compound. These results show that the thermal gelation problem of GMA-ESBR can be solved by reacting an epoxy group with a silanol group on the silica surface using WMB technology. If GMA-ESBR, which is a functionalized ESBR, is used in the tread compound for passenger cars, it will be able to produce tires with excellent abrasion and rolling resistance.

## Figures and Tables

**Table 1:** Formulation for the ESBR and GMA-ESBR polymerization (gram).

Ingredients	Name	ESBR	GMA-ESBR
Monomers	Styrene	112	110.8
	Butadiene	288	288
	Glycidyl methacrylate	-	1.2
Chain transfer agent	*tert*-Dodecyl mercaptan	0.69	
Solvent	Distilled water	640	
Surfactants	Rosin soap	39.5	
	Fatty soap	100.1	
Catalysts	EDTA	0.1	
	FES	1	
	SFS	0.2	
Initiator	*p*-Menthane hydroperoxide	0.5	

**Table 2:** Experimental formulation for rubber compounds (phr).

Items	ESBR DMB	GMA-ESBR DMB	GMA-ESBR WMB
ESBR	100	-	-
GMA-ESBR	-	100	-
GMA-ESBR WMB^1^	-	-	188
TESPT 10 wt % modified silica (7000GR)^2^	88	88	-
Zinc oxide	3		
Stearic acid	2		
6PPD	1		
Sulphur	1.5		
CBS	1.5		
DPG	1.5		

^1^ GMA-ESBR 100 phr, TESPT 10 wt % modified silica 88 phr. ^2^ TESPT 10 wt % modified silica 88phr; 80 phr silica was modified with 8 phr silane (TESPT).

**Table 3:** Mixing procedures of SMB and FMB.

First stage for silica masterbatch (SMB)	
Time (min:sec)	Action
0:00	Add rubber, WMB
0:40	Add 12 silica, coupling agent
1:40	Add 12 silica, coupling agent
2:40	Add ZnO, St/A, 6PPD
4:40	Ram up
12:00	Dump
Second stage for final masterbatch (FMB)	
0:00	Add SMB
0:20	Add curatives
2:00	Dump

**Table 4:** Characteristics of ESBR and GMA-ESBR.

Sample Name	Molecular Weight (*M_W_*)	Poly Dispersity Index (PDI)	Styrene (wt %)	Vinyl Content (% in Butadiene)	GMA Content (wt %)	Gel Content (wt %)	Mooney Viscosity (M*L*_1+4_ 100 °C)
ESBR	672,000	3.2	26	20	0	0	93
GMA-ESBR	720,000	4.5	25.5	20	2.5	0	124

**Table 5:** Gel contents after thermal treatment (5 min, at 100 °C).

Gel Contents	ESBR	GMA-ESBR
Raw polymer	0%	0%
Raw polymer after heating (100 °C)	0%	54%

**Table 6:** The Payne effect (ΔG′) of the ESBR DMB, GMA-ESBR DMB, and GMA-ESBR WMB compounds.

Sample Code	ESBR DMB	GMA-ESBR DMB	GMA-ESBR WMB
ΔG′	1197	1173	861

**Table 7:** Cure characteristics and crosslink densities of ESBR and GMA-ESBR compounds.

Sample Code	Unit	ESBR DMB	GMA-ESBR DMB	GMA-ESBR WMB
*t*_10_	min:sec	2:14	2:16	2:00
*t*_90_	min:sec	8:48	10:10	12:04
*T*_min_	N-m	0.37	0.39	0.38
*T*_max_	N-m	1.46	1.44	1.40
Crosslink density	10^−4^mol/g	0.80	0.94	0.88

**Table 8:** Mechanical and abrasion properties of ESBR and GMA-ESBR vulcanizates.

Sample Code	Unit	ESBR DMB	GMA-ESBR DMB	GMA-ESBR WMB
*M*_100%_	MPa	2.01	2.02	1.84
*M*_300%_	MPa	8.24	9.22	9.55
*M*_300%_/*M*_100%_		4.1	4.5	5.2
Elongation	%	618	547.6	507
DIN abrasion	mg	138	126	111

**Table 9:** Viscoelastic properties of ESBR DMB, GMA-ESBR DMB, and GMA-ESBR WMB compounds.

Sample Code	ESBR DMB	GMA-ESBR DMB	GMA-ESBR WMB
T_g_	−31.3	−29.7	−30.1
Tan δ at T_g_	0.8331	0.8451	0.8890
Tan δ at 0 °C	0.238	0.239	0.231
Tan δ at 60 °C	0.123	0.120	0.108
